# Aiming for spatial and temporal control of gene expression

**DOI:** 10.7554/eLife.107945

**Published:** 2025-07-10

**Authors:** Stanislao Igor Travisano, Ching-Ling Lien

**Affiliations:** 1 The Saban Research Institute of Children's Hospital Los Angeles Los Angeles United States; 2 The Saban Research Institute of Children's Hospital Los Angeles, Departments of Surgery, Stem Cell Biology and Regenerative Medicine, Keck School of Medicine, University of Southern California Los Angeles United States

**Keywords:** bioluminescent imaging, gene therapy, tissue regeneration enhancers, adeno-associated virus, myocardial infarction, cardiac, Mouse

## Abstract

Bioluminescent imaging is helping researchers better understand the effectiveness of tissue regeneration enhancers delivered to injured heart tissue by different adeno-associated virus vectors.

**Related research article** Wolfson DW, Hull JA, Li Y, Gonzalez TJ, Jayaram MD, Devlin GW, Cigliola V, Oonk KA, Rosales A, Bursac N, Asokan A, Poss KD. 2025. Spatial and longitudinal tracking of enhancer-AAV vectors that target transgene expression to injured mouse myocardium. *eLife* **14**:RP107148. doi: 10.7554/eLife.107148.

Gene therapy – where functional copies of genes are delivered to certain cells in a patient – is becoming a reality for patients with cancer and a range of genetic disorders, including Duchenne muscular dystrophy and sickle cell disease. Such therapies are often delivered via an adeno-associated virus, a generally non-toxic viral vector that has shown promise as a delivery system in various gene therapy trials (Kuzmin et al., 2021; Wang et al., 2019).

This delivery system also has the potential to drive the expression of repair genes in injured tissues by delivering short sequences of DNA called TREEs (which is short for tissue regeneration enhancer elements). TREEs are activated by injury signals and can drive the expression of regenerative genes in damaged and regenerating tissues. Although TREEs were discovered in zebrafish, which have the capacity to regenerate damaged tissue, they can also trigger the expression of repair genes in other species. In particular, three enhancers that direct gene expression during heart regeneration in zebrafish (Kang et al., 2016; Goldman et al., 2017) can, when delivered using adeno-associated viruses, improve heart repair in both mice and pigs (Yan et al., 2023).

However, translating this strategy into clinical use is challenging, with a major concern being the risk of off-target effects, particularly when using genes that stimulate cell proliferation. Now, in eLife, Aravind Asokan, Kenneth Poss and colleagues – including David Wolfson as first author – report how different adeno-associated virus capsids and TREEs affect gene expression in a mouse model of myocardial infarction (Wolfson et al., 2025). The team – who are based at Duke University, Vanderbilt University, the Morgridge Institute for Research, and the University of Wisconsin-Madison – used bioluminescent imaging to track the expression of TREE-activated reporter genes in both time and space, and to explore the influence of the different delivery systems.

The expression patterns of two TREEs associated with heart injury (REN and 2ankrd1aEN) were analysed using bioluminescent imaging following delivery via AAV9 – a widely used adeno-associated virus variant known for driving high gene expression in the heart, but with notable off-target effects in the liver (Prasad et al., 2011). Wolfson et al. also delivered the same TREEs using AAV.cc84 – an engineered AAV9 variant specifically designed to reduce off-target effects in the liver (Gonzalez et al., 2023; Gonzalez et al., 2022). This revealed that AAV.cc84 achieved effective cardiac delivery with markedly reduced off-target expression compared to AAV9.

While AAV9 targets heart muscle cells well, it tends to deliver genes mostly to cells bordering the injury, rather than those in the damaged area (Konkalmatt et al., 2012). To overcome this limitation, Wolfson et al. screened adeno-associated virus capsid libraries to find variants that deliver TREE- directed reporters to the heart muscle cells within the injury site while scar tissue is forming. A new capsid variant – AAV.IR41 – showed an enhanced ability to deliver TREE-reporters to cells within the damaged area.

The findings of Wolfson et al. highlight the potential of combining injury-induced TREEs with specially designed viral carriers to control gene expression at specific locations and times after tissue injury to promote regeneration. Despite these advancements, several challenges remain. While in vivo bioluminescence imaging is powerful, it is constrained by tissue depth limitations, restricting its use in larger animal models and therefore necessitating complementary imaging or molecular approaches to validate findings across species.

Although the off-target expression observed in areas such as the liver, neck and abdomen can be partially minimised by capsid design, a deeper understanding of how different gene elements interact, as well as why different viral vectors are specific to certain tissues, is required. Reporter genes delivered by AAV.IR41 showed overall much stronger expression than AAV9 and therefore off-target effects could be more widespread. Combining features of AAV.cc84 and AAV.IR41 could represent an exciting opportunity to further minimize such off-target effects.

Furthermore, the efficacy of AAV.IR41 could be better understood by comparing how it works after different types of heart injury: ischemia-reperfusion (where the blood flow is restored after the blockage) and injuries where the blockage is permanent. Does the efficacy of AAV.IR41 improve when blood flow is restored by reperfusion? Lastly, it will be interesting to determine whether TREE-driven gene expression returns to baseline once heart regeneration is complete, which would suggest that TREEs are only active during injury and become inactive after recovery.

Looking forward, further optimization of TREE-directed gene delivery via adeno-associate virus vectors could pave the way for more therapeutic applications. Translating such strategies into larger animal models and, ultimately, clinical settings will require scalability, new imaging methods, and robust safety assessments. The work of Wolfson et al. establishes a critical platform for the rational design of enhancer-adeno-associated virus combinations tailored to the tissue regeneration, and lays the groundwork for a new generation of gene therapies aimed at harnessing the body’s intrinsic repair mechanisms.
